# Unbalanced intakes of sodium and potassium among Tunisian adults: A cross‐sectional study

**DOI:** 10.1002/fsn3.2197

**Published:** 2021-02-26

**Authors:** Radhouene Doggui, Jalila El Ati, Sonia Sassi, Houda Ben Gharbia, Ayoub Al‐Jawaldeh, Myriam El Ati‐Hellal

**Affiliations:** ^1^ INNTA (National Institute of Nutrition and Food Technology) SURVEN (Nutrition Surveillance and Epidemiology in Tunisia) Research Laboratory Tunis Tunisia; ^2^ University of Tunis El Manar Tunis Tunisia; ^3^ World Health Organization (WHO) Regional Office for the Eastern Mediterranean (EMRO) Cairo Egypt; ^4^ Laboratory Materials Molecules and Applications LR11ES22 Tunis Tunisia; ^5^ IPEST University of Carthage Tunis Tunisia

**Keywords:** cross‐sectional study, diet, Eastern Mediterranean region, linear model, salt, Tunisia

## Abstract

The prevention and management of hypertension are untimely associated with a lowering of sodium intake. The present study aimed to evaluate the sodium and potassium intake levels of Tunisian population through measurement of 24‐hr urinary sodium excretions. A randomly, multistage, cross‐sectional study was conducted in an urban region (Bizerte) in Tunisia during 2015. The target population involved adults aged from 25 to 64 years. Sodium, potassium, and creatinine concentrations were determined in each urine sample using indirect potentiometric method. From the 420 selected participants, only 194 gave urine samples complying completeness criteria. A multivariate regression model was used to assess the variables related to sodium and potassium excretion. The daily mean excretion of sodium and potassium was 138.3 ± 46.5 mmol/d (corresponding to 8.1 ± 2.7 g/d of salt intake) and 61.0 ± 22.7 mmol/d, respectively. More than 87.1% of the participants (89.8% for men vs. 84.9% for women; *p = *.31) exceeded the WHO recommendation of 5 g/d. The upper limit of 10 g salt intake per day was still exceeded by 26.3%. After adjusted analysis, sex (for women, coef = −1.6; (95% CI: −2.4, −0.7)), level of instruction (≥30 kg/m^2^, coef = +1.1; (95% IC: 0.4–2.0)), and body mass index (≥30 kg/m^2^, coef = +1.1; (95% CI: 0.1, 2.0)) were associated with the sodium excretion. High sodium intake and inadequate potassium intake were found among participants. This consumption profile complies with the diet westernization context occurring in Tunisia. The initiated strategy focused on the downward of sodium in bread (the main source of salt intake) seems to be promising.

## Graphical abstract

Excess of salt intake is one of the major dietary risk factor of chronic diseases. In the following study, we are assessing salt intake using both urinary sodium excretion and dietary assessment among Tunisian adults. Of the food groups, bread is the most important contributor to the daily salt intake.

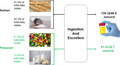

## 1 INTRODUCTION

According to the Global Burden of Disease study (GBD) 2017, high intake of sodium accounts for 3 million deaths and 70 million disability‐adjusted life years (Afshin et al., 2019). High sodium intake is a risk factor for several chronic diseases, that is, cardiovascular impairments (Brown et al., 2009; Ha, 2014; He & MacGregor, 2009) but also with several chronic pathologies such as stomach cancer (D'Elia et al., 2014; Peleteiro et al., 2011), osteoporosis (Park et al., 2016), kidney diseases (Boero et al., 2002), and asthma (Mickleborough, 2010). The World Health organization member states have agreed the WHA 66.10 Resolution to reduce the salt intake to less than 5 g per day (relatively 30% by 2025) (House, 2013).

Tunisia, as an example of the Eastern Mediterranean region, has experienced the nutrition transition during the last decades as claimed by several reports (Abassi et al., 2019; Aounallah‐Skhiri et al., 2011; Romdhane et al., 2002; Traissac et al., 2016). Increase of salt intake is closely related to the nutrition transition (Popkin et al., 2012), and Tunisian population seems to fit to this rule according to the prediction made by *Mason and al*. (≈14 g/d) (Mason et al., 2014; WHO & FAO expert, 2003). Other countries from the Eastern Mediterranean region (e.g., Bahrain, Egypt, Iran, Joran) have shown high consumption of salt (>5 g per day) (Al Jawaldeh et al., 2019). Regarding the challenge to reduce salt intake, accurate measurement of salt intake is crucial to direct the intervention both at the baseline and for the follow‐up for interventional study design. Various approaches have been proposed to assess salt intake, that is, dietary methods (24‐hr dietary recall, food record, and food frequency questionnaire) and biological methods (e.g., 24‐hr urinary sodium excretion, casual urine). The 24‐hr urinary excretion of sodium is the recommended method for assessing sodium (salt) intake among individuals (Cogswell et al., 2015).

Potassium was documented extensively as a modifier of the association between excess sodium (salt) intake and occurrence of cardiovascular damages (Aburto et al., 2013; Ando et al., 2010; D'Elia et al., 2014). Moreover, the potassium increases the sodium urinary excretion which contributes to harm reduction of excessive sodium intake (Mercado et al., 2018). The World Health Organization recommends that adults should consume ≥90 mmoL per day (WHO, 2012). The 24 hr is the recommended method for the assessment of potassium intake.

However, collecting 24‐hr urine specimens is often not feasible and survey based on have low participation rate which leads to biased estimates (Hawkes & Webster, 2012). The 24‐hr dietary recall remains an important tool for public health policy follow‐up especially when the urine collection is not feasible. Moreover, the 24 hr will allow the identification of the food group contributors of nutrient intake.

Until now, no study has been conducted among the Tunisia neither for sodium nor for potassium based on the 24‐hr urine specimen collection.

The current study aimed to estimate the average intakes of sodium (a proxy of salt consumption) and potassium levels among Tunisian adults; secondly, to identify factors associated with the urinary sodium excretion and food group contribution to the daily intake; and finally, to measure the agreement between nutrients in 24‐hr urinary excretion and 24‐hr dietary recall.

## 2 METHODS

### 2.1 Study area

Bizerte is an urban city located in the northeast of Tunisia. This city was selected as a pilot area for implementing the strategy to prevent and control obesity.

### 2.2 Study design and subjects

The survey was cross‐sectional, conducted during January 2015 among Tunisian adults aged 25–64 years. The sampling frame was derived by the Tunisian National Institute of Statistics from the database of the census of the population carried out in 2014. Stratification was according to the three delegation of Bizerte City. At the first stage, 21 census districts were randomly selected, with a probability proportional to size in number of eligible households (i.e., featuring at least then two 25‐ to 65‐year‐old subjects). At the second stage, 10 eligible households were randomly sampled in each district. The third stage of selection was performed during the implementation of the field survey: In each household, two subjects (1 male and 1 female) from the targeted age range were selected at random, from the list resulting from the enumeration of all household members. Individuals with a diagnosis of diabetes, hypertension, or renal disease, or who had been prescribed diuretics, were excluded. Pregnant women were also excluded from the protocol. A total of 420 subjects were selected to participate.

### 2.3 Urine collection

For every respondent, three visits were planned: *day 1*—the interviewer gave each participant two graduated glass containers, one to use at home and the other for urine collections out of home. The interviewer provided to each participant the instructions for collecting 24‐hr urine samples (instructions: from the second day, start collecting 24‐hr urine after throwing the first urination of the day and noting the time; do not forget to note the end time of the collection); *day 2*—the interviewer reinforced the instructions for the proper collection of the 24‐hr urine sample; and *day 3*—participants provided their 24‐hr urine samples to the interviewers. When the collection of the 24‐hr urine sample had to be repeated, further meetings were planned.

### 2.4 Urine analysis

Urine samples were sent to the Clinical Biology Laboratory of the National Institute of Nutrition and Food Technology. After measuring the total volume, an aliquot of 5 ml was withdrawn from every urine sample. Sodium (Na), potassium (K), and creatinine content for 24‐hr urine were then quantified. Na and K were measured using an indirect potentiometric method with selective solid membranes for each ion, connected to an autoanalyzer DX800 (Beckman Coulter, Northwell, Luton, England). The between‐day precision was CV = 4.2% for urinary sodium and 3.3% for urinary potassium. Urinary creatinine was determined according to the modified Jaffe reaction, and the between‐day precision was 5.9%. The precision of our techniques complies with the desirable specifications (Ricos et al., 1999).

To confirm the proper collection of 24‐hr urine, all samples with urinary creatinine < 4 mmol/d for women, or < 6 mmol/d for men, or with a total volume less than 500 ml for both sex were excluded (Land et al., 2014). The 24‐hr sodium excretion content (mmol/d) was calculated using the formulation:

1 mmol Na = 0.0585 g NaCl

1 g NaCl = 1/0.0585 Na = 17 mmol Na

To convert potassium from mmol to mg, we multiplied the values by 39.1.

For the purpose of measuring agreement between 24‐hr urinary data and dietary recall methods, urinary values were divided by 0.77 and 0.86 to account for external losses of potassium and sodium, respectively (Holbrook et al., 1984).

### 2.5 Covariates

As well as a 24‐hr urine sample collection, a self‐administered questionnaire was fulfilled by each participant, assisted by a trained investigator, including sociodemographic information (age, sex, level of education, occupation, number of children, marital status, menopausal status for women) and anthropometric measurements.

Anthropometric measurements were performed as follows: height was measured by stadiometer (Person‐check®, Kirchner & Wilhelm, Germany), and weight was measured to 100 g on a calibrated scale (Detecto, USA). To assess the overall obesity, body mass index (BMI) was calculated using the following formula: BMI = weight (kg)/height (m)^2^. According to WHO, the principal cutoff points are as follows: BMI < 18.5 kg/m^2^ indicates underweight, BMI within 18.5 – 24.9 indicates normal range, BMI ≥ 25 kg/m^2^ indicates overweight, and BMI ≥ 30 kg/m^2^ indicates obesity (WHO, 2000).

### 2.6 Dietary intake

The 24‐hr recall was used to obtain a quantitative food intake. The method consists of a face‐to‐face interview, conducted by a trained dietician, during which the interviewee was asked to provide detailed information about everything she/he had to drink and eat over the day of urine collection. A detailed and precise description of each food and beverage consumed (including food preparation and cooking methods, brand name of commercial products) was collected. Estimates of the amount of food and drinks item consumed were obtained using household measures or food photographs. A specific Tunisian food composition database (El Ati et al., 2007) and the Food Processor software (Inc.) were used to compute average daily intake of energy (kcal/d), and macro‐ and micronutrients. Extreme and likely inaccurate records (energy intake ≥95th percentile of the observed distribution or less than the estimated basal metabolic rate) were excluded from the analyses (Goldberg et al., 1991; Livingstone & Black, 2003). Salt intake (g/d) was calculated from sodium intake (g/d × 2.542). The adequacy of energy, macro‐ and micronutrient intake was evaluated with regard to the WHO recommendation (WHO & FAO expert, 2003). The recommended daily sodium (or salt) intake is below 2 g/d (or 5 g of salt/d), and the tolerable upper limit dose is 10 g/d. The recommended daily potassium intake suggested by the WHO experts is about at least 90 mmol/d (WHO, 2012). The sodium‐to‐potassium ratio should be close to 1.0 (WHO & FAO expert, 2003).

Based on data obtained during a previous study (*Obe‐Maghreb* project (NIH, 2013)) and our knowledge about the Tunisian dietary habits, we recognize 15 food groups (bread whole meal; cereals and pasta; eggs; fishes and shellfish; fruits; legumes; meats; milk and dairy products; nuts and seeds; oils and other fats; sugars and confectionary; tea, coffee, and water; vegetables; and white breads; meats).

### 2.7 Data management and analysis

Epidata 3.1 software^®^ was used for data entry. The global statistical analyses were realized using Stata 16.0 software ®. All continuous variables were expressed as mean ± standard deviation and median (where it was necessary), while categorical variables were expressed as percentage. After the check of variance homogeneity, means comparison was operated using *t* test of Student, else Mann–Whitney test was used. Student's *t* test (or Kruskal–Wallis test) was used to compare means across more than two groups. Chi‐squared test was used to assess the difference as regards the distribution of categorical variables.

The Bland–Altman plot and Passing Bablok regression were used to assess the agreement between 24‐hr dietary recall and urinary excretion methods (Bland & Altman, 1986). For Passing Bablok regression, if the 95% confidence interval (CI) of the intercept and slope includes 0 and 1 means that there are neither systematic nor proportional differences between both methods, respectively (Bablok & Passing, 1985).

## 3 RESULTS

### 3.1 Population characteristics

The response rate was as high as 46.2%. Table 1 shows the characteristics of the study sample. Mean age value of men was significantly higher than that of women (46.4 years vs. 43.3 years, *p* =.049). A significant gender difference was found for height, weight, and hip circumference. “Not working” factor was less frequent among men (*p* <.0001) and who achieved higher educational attainment (*p* =.001).

**TABLE 1:** Tunisian adults by physiological, anthropometric, nutritional, and socioeconomic features (*n* = 194)

	Men (*n* = 88)	Women (*n* = 106)	Total (*n* = 194)	*p*‐value Men versus women
Anthropometric characteristics				
Age (years)	46.4 ± 0.9^a^	43.3 ± 1.2	44.7 ± 0.8	.049
Height (cm)	169.8 ± 1.0	158.7 ± 1.1	163.7 ± 1.1	<.0001
Weight (kg)	80.2 ± 1.7	75.7 ± 1.7	77.8 ± 1.2	.038
Waist circumference (cm)	98.9 ± 1.5	99.3 ± 1.7	99.1 ± 1.1	.576
Hip circumference (cm)	105.5 ± 1.2	108.1 ± 1.4	106.9 ± 0.9	.006
Body mass index (kg/m^2^)	27.9 ± 0.6	32.0 ± 2.5	30.1 ± 1.4	.925
Overweight (%, including obesity)	65.9	78.3	72.7	.054
Obesity (%)	29.6	46.2	38.7	.018
Waist‐to‐height ratio	0.6 ± 0.01	0.6 ± 0.01	0.6 ± 0.01	.005
Marital status (%)				
No	11.4	10.4	10.8	.826
Yes	88.7	89.6	89.2	
Professional activity (%)				
Yes	85.2	21.7	50.5	<.0001
No	14.8	78.3	49.5	
Level of instruction (%)				
No formal schooling	4.6	13.3	9.4	.001
Primary school	25.3	43.8	35.4	
Secondary or more	70.1	42.9	55.2	

^a^ Mean ± standard error of deviation.

### 3.2 Distribution of urinary parameters and gender difference

**FIGURE 1:**
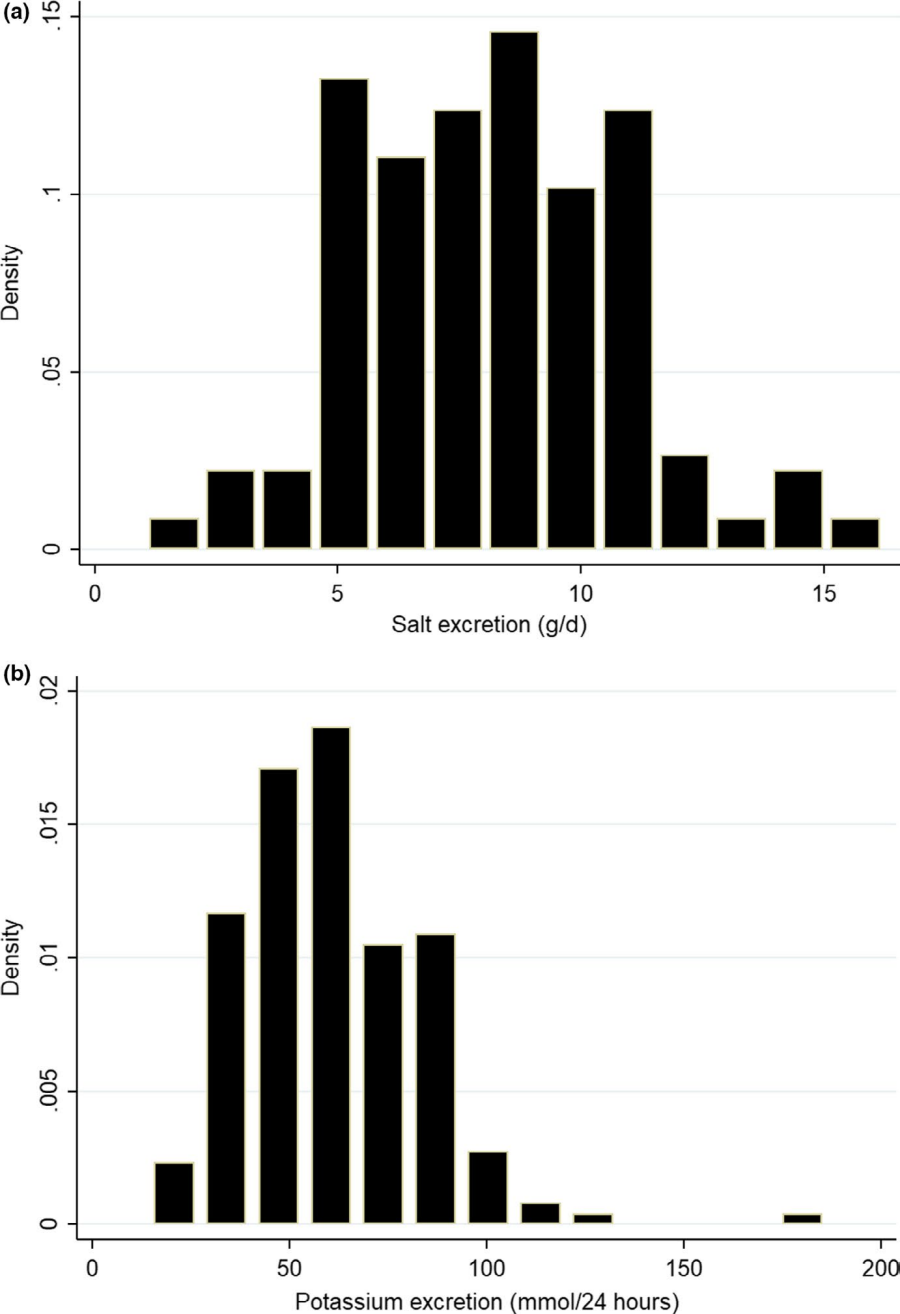
Histogram of the individual data distribution of 24‐hr urinary excretion of salt (a) and potassium (b)

The urine volume, creatinine concentration, potassium‐to‐creatinine, sodium‐to‐creatinine, and sodium‐to‐potassium ratios were not normally distributed and skewed on the right (*p* <.001). The log transformation of these variables did not deliver a better fit to normal distribution justifying the use of nonparametric test for medians comparison. As shown in Table 2, the coefficient of variation (CV) of urine volume was 43.9% for men versus 45.2% for women. Wide dispersion was also found for creatinine values (40.8%) over all samples. The narrowest dispersion was showed for sodium‐to‐creatinine ratio with an average CV of 39.2% (49.2% for men vs. 28.1% for women). Mean value of urine volume was equally according to gender; however, as expected, creatinine excretion was found to be higher among men (*p* <.0001). Urinary sodium and potassium distributions are displayed in Figure 1.

**TABLE 2:** Biochemical features according to gender

	Men (*n* = 88)			Women (*n* = 106)			Total (*n* = 194)			*p*‐value Men versus Women
	Mean ± SD^a^	Median (interquartile range)	CV (%)	Mean ± SD^a^	Median (interquartile range)	CV (%)	Mean ± SD^a^	Median (interquartile range)	CV (%)	
Volume (mL)	1,140.0 ± 500.6	1,010.0 (800 – 1,350)	43.9	1,146.9 ± 518.1	1,000.0 (720.0 – 1,500.0)	45.2	1,143.8 ± 508.9	1,000.0 (750.0 – 1,400.0)	44.5	.926
Creatinine (mmol/L)	13.4 ± 4.4	12.8 (9.8 – 16.7)	38.6	10.8 ± 4.3	10.4 (7.4 – 13.2)	39.9	12.0 ± 4.9	11.8 (7.9 – 15.0)	40.8	<.0001
Na (mmol/d)	150.8 ± 49.3	144.5 (114.0 – 181.3)	32.7	128.0 ± 41.6	130.0 (98.8 – 160.7)	32.5	138.3 ± 46.5	139.4 (102.9 – 172.6)	33.6	.001
K (mmol/d)	66.3 ± 20.5	63.9 (53.0 – 82.3)	30.9	56.7 ± 23.6	53.1 (43.9 – 67.2)	41.6	61.0 ± 22.7	59.9 (44.7 – 75.2)	37.2	.003
Na:K	2.4 ± 0.9	2.3 (1.9 – 2.8)	37.4	2.4 ± 0.8	2.3 (1.9 – 2.9)	34.6	2.4 ± 0.9	2.3 (1.9 – 2.9)	35.8	.950
Na:creatinine ratio	11.6 ± 4.0	10.6 (9.0 – 13.5)	34.8	12.3 ± 4.5	11.9 (9.6 – 14.7)	36.3	12.0 ± 4.3	11.3 (9.3 – 14.1)	35.7	.267
K:creatinine ratio	5.3 ± 2.6	4.4 (3.7 – 6.0)	49.2	5.2 ± 1.5	5.1 (4.1–5.9)	28.1	5.3 ± 2.1	4.9 (3.9 – 5.9)	39.2	.731

Abbreviation: CV, coefficient of variation.

^a^ Mean ± standard error of deviation.

### 3.3 Salt excretion measures based on urinary sodium excretion

Assuming that the urinary excretion of sodium (150.8 ± 49.3 mmol/d) comes from the diet, the total salt intake from the entire population was amounted to 8.1 ± 2.7 g/d. The sodium excretion was higher among men; however, this difference did not stand after adjusting to creatinine excretion (Table 3). Participants with occupational status have higher sodium excretion 146.5 versus 129.9 mmol/d (*p* =.013) corresponding to 8.6 versus 7.6 g/d of salt, respectively. More than 87.1% of the participants (89.8% for men vs. 84.9% for women; *p* =.31) exceeded the WHO recommendation of 5 g/d. The upper limit of 10 g salt intake per day was still exceeded by 26.3% (34.1% for men vs. 19.8% for women; *p* =.024).

**TABLE 3:** Multivariate regression models, salt excretion, and prevalence of excess intake by sociodemographic and anthropometric categories

	*n*	Urinary sodium (mmol/d)	Salt excretion (g/d)	Unadjusted analysis^a^		Adjusted analysis^b^		Prevalence of excessive salt intake			
				Diff ^c^	95% CI^d^	Diff ^c^	95% CI ^d^	>5 g/d ^e^	>6 g/d	>8 g/d	>10 g/*df*
Gender		*P* ^g^ = .001		*P* ^h^ = .001		*P* ^h^ < .0001	*P* ^i^ = .31	*P* ^i^ = .20	*P* ^i^ = .030	*P* ^i^ = .024	
Men	88	150.8 ± 49.3^j^	8.8 ± 2.8	‐		‐		89.8	79.6	60.2	65.9
Women	106	128.0 ± 41.6	7.5 ± 2.4	−1.3	−2.1, −0.6	−1.6	−2.4, −0.7	84.9	71.7	44.3	34.1
BMI (kg/m^2^)		*p* =.40		*p* =.40		*p* =.10	*p* =.20	*p* =.28	*p* =.91	*p* =.72	
< 25	53	132.0 ± 41.2	7.7 ± 2.4	‐		‐		86.8	69.8	49.1	28.3
≥25 & <30	66	137.7 ± 50.9	8.1 ± 3.0	0.3	−0.7, 1.3	0.6	−0.4, 1.5	81.8	72.7	53.0	22.7
≥30	75	143.3 ± 46.1	8.4 ± 2.7	0.7	−0.3, 1.6	1.1	0.1, 2.0	92.0	81.3	52.0	28.0
Age (years)		*p* =.50		*p* =.50		*p* =.30	*p* =.30	*p* =.76	*p* =.48	*p* =.96	
24–34	38	131.8 ± 38.4	7.7 ± 2.2	‐		‐		81.6	73.7	42.1	29.0
35–44	55	142.8 ± 48.9	8.4 ± 2.9	0.6	−0.5, 1.8	0.5	−0.7, 1.7	90.9	80.0	56.4	25.5
45–54	71	141.6 ± 49.8	8.3 ± 2.9	0.6	−0.5, 1.7	0.0	−1.1, 1.2	90.1	74.7	54.9	26.8
>55	30	130.8 ± 43.5	7.7 ± 2.5	−0.1	−1.4, 1.3	−0.7	−2.0, 0.7	80.0	70.0	46.7	23.3
Marital status		*p* =.74		*p* =.74		*p* =.212	*p* =.84	*p* =.67	*p* =.40	*p* =.80	
Yes	173	137.9 ± 46.8	8.1 ± 2.7	‐		‐		87.3	75.7	52.6	26.3
No	21	141.5 ± 45.6	8.3 ± 2.7	0.2	−1.0, 1.5	0.8	−0.5, 2.1	85.7	71.4	42.9	28.6
Professional activity		*p* =.013		*p* =.012		*p* =.157	*p* =.016	*p* =.081	*p* =.062	*p* =.042	
Yes	98	146.5 ± 48.4	8.6 ± 2.8	‐		‐		92.9	80.6	58.1	32.7
No	96	129.9 ± 43.2	7.6 ± 2.5	−1.0	−1.7, −0.2	−0.6	−1.5, 0.2	81.3	69.8	44.8	19.8
Level of instruction		*p* =.12		*p* =.12		*p* =.020	*p* =.41	*p* =.34	*p* =.11	*p* =.12	
No formal schooling	18	130.4 ± 48.2	7.6 ± 2.8	−0.2	−1.6, 1.2	0.3	−1.1, 1.7	77.8	61.1	38.9	33.3
Primary school	68	147.8 ± 50.5	8.6 ± 3.0	0.8	−0.01, 1.6	1.2	0.4, 2.0	89.7	77.9	61.8	33.8
Secondary or more	106	133.9 ± 43.4	7.8 ± 2.5	‐		‐		86.8	75.5	48.1	20.8

^a^ Unadjusted analysis: association of each covariate with salt intake in g/day.

^b^ Adjusted analysis: multivariate model for salt intake including socioeconomic and anthropometric characteristics of subjects.

^c^ Unadjusted or adjusted difference between category and reference category as regards the salt intake.

^d^ *p* = .95 confidence interval.

^e^ WHO recommendation for salt intake.

^f^ Upper limit for salt intake.

^g^ Unadjusted or adjusted *p*‐value for comparison of urinary sodium excretion (or corresponding salt intake) means between categories of socioeconomic and anthropometric characteristics of subjects.

^h^ Unadjusted or adjusted *p*‐value for association of salt intake between categories of socioeconomic and anthropometric characteristics of subjects.

^i^ *p*‐value for comparison of percentage of excess salt intake based on different cutoffs.

^j^ Geometric mean ± standard deviation.

### 3.4 Relationship between sodium intake and covariates

In Table 2, considering the absolute values, men had the highest mean of salt intake (8.8 vs. 7.5 g/d; *p* =.001).

In unadjusted analyses, a significant difference (*p* <.0001) of −1.3 g/d of salt intake appears for women in comparison with men. This difference was increased after adjustment to covariates (−1.6 g/d; *p* <.0001). However, in adjusted analyses, a significant difference of sodium was associated with obesity (+1.1(95% CI: 0.1–2.0) g/d) and lower level of instruction (+1.2 (95% CI: 0.4–2.0) g/d for primary instruction level).

### 3.5 Urinary potassium excretion and association with covariates

The average daily excretion of potassium was 61.0 ± 22.7 mmol/d (66.3 ± 20.5 vs. 56.7 ± 23.6 mmol/d; *p* =.0003). About 10.3% of participant met the WHO recommendations for potassium intake, and a sex difference was reported (14.8% for men vs. 6.6% for women; *p* =.062). Both crude and adjusted regression models (detailed data not shown) revealed that potassium excretion was lower among women (coef. =−11.2, t=−3.0, *p* =.0003). However, no significant association was found with age, BMI, matrimonial status, professional activity (classified as employed or unemployed), and instruction level. The sodium‐to‐potassium ratio did not differ according to gender (10.6 for men vs. 2.43 for women; *p* =.95).

### 3.6 Dietary intake data

The mean daily intake of energy and macronutrient is shown in Table 4. A high contribution of proteins and free sugar to the daily energy intake was found while low total carbohydrates and dietary fibers intake. The rest of macronutrient energy profiles reflects a dietary pattern in line with dietary goals established by the WHO.

**TABLE 4:** Energy and macronutrient profile of the Tunisian adults (*n* = 194)

Macronutrients	Crude daily intake	Contribution to the daily energy intake (%)	Recommended dietary intake (% of total energy)^a^
Energy (kcal/day)	1914.3 ± 38.2^b^	‐	‐
Protein (g/day)	76.4 ± 2.2	16.0	10–15
Carbohydrates (g/day)	247.7 ± 5.2	51.8	55–75
Free sugar (g/day)	61.9 ± 31.0	12.9	<10
Total fat (g/day)	64.0 ± 1.9	30.0	15–30
Saturated fatty acids (%)	16.7 ± 0.7	7.9	<10
Polyunsaturated fatty acids (%)	19.2 ± 0.6	9.0	6–10
n−3 Polyunsaturated fatty acids	2.0 ± 0.1	1.0	1–2
n−6 Polyunsaturated fatty acids	15.3 ± 0.5	7.1	5–8
Monounsaturated fatty acids	22.6 ± 0.8	10.6	‐
Dietary fiber (g/day)	21.0 ± 0.6	‐	≥25 g/day
Cholesterol (mg/day)	158.7 ± 10.9	‐	<300 mg/day
Salt (g/day)	10.6 ± 2.8	‐	<5 g/day
Sodium (mg/day)	2,643.7 ± 915.0	‐	2000 mg/day

^a^ WHO recommendations.

^b^ Mean ± standard deviation.

### 3.7 Sodium intake measures based on diet consumption estimation

Overall, the mean and median values of sodium intake were 4,251.8 ± 1,113.2 mg/d and 4,115.2 mg/d corresponding to 10.6 g/d and 10.3 g/d of salt, respectively. Salt intake was not significantly higher for men than women (11.3 ± 3.0 g/d vs. 10.3 ± 2.5 g/d; *p* <.092) even after sodium adjustment to the daily energy intake (5.8 vs. 6.0 g/1000 kcal; *p* =.505).

### 3.8 Sodium and potassium food sources

**FIGURE 2:**
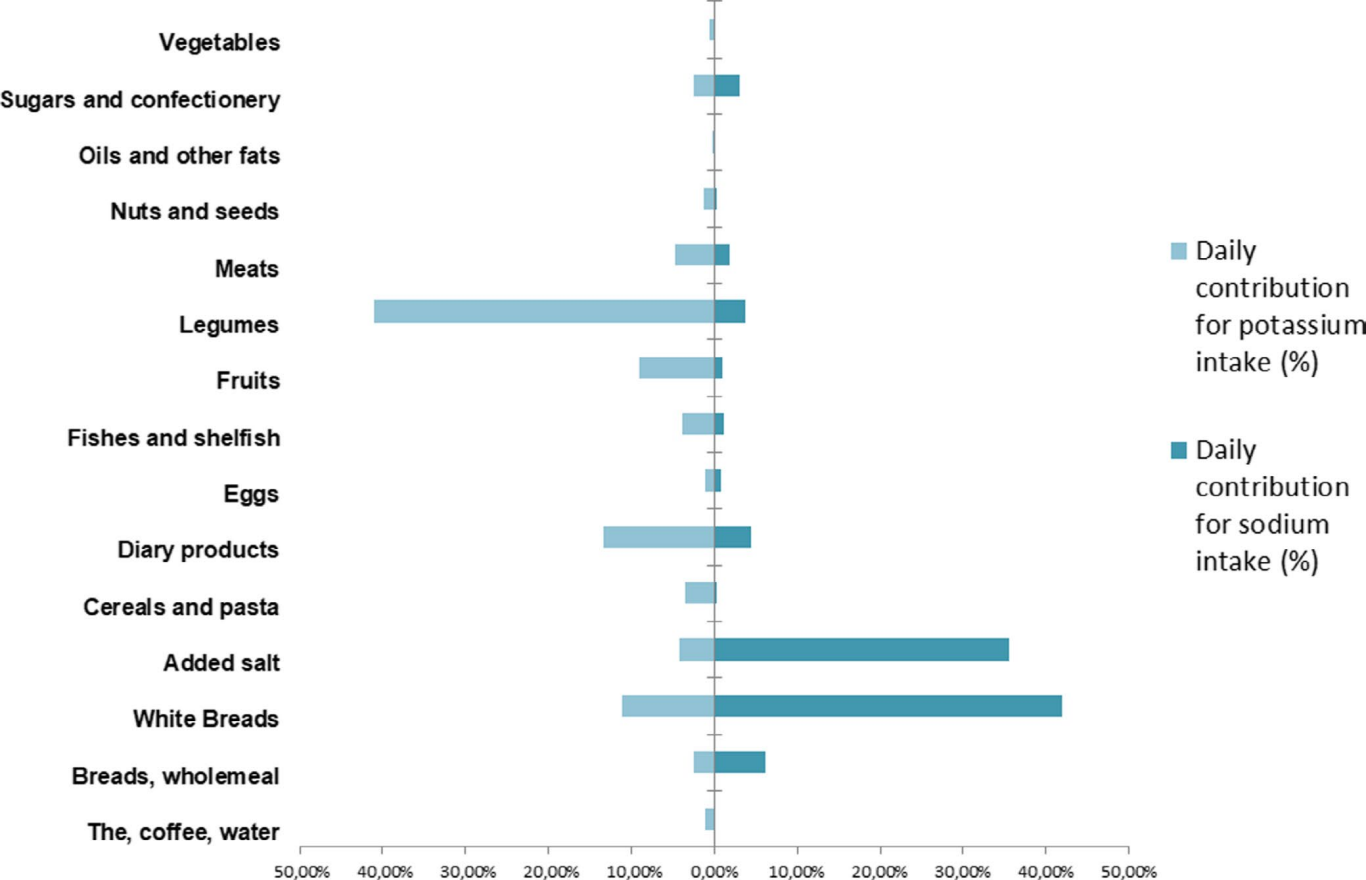
Food group contribution (%) to the daily sodium (dark blue) and potassium (light blue) intakes

The daily contribution of food groups to the total sodium and potassium intakes is presented in Figure 2. The main contributors to the daily sodium intake were white breads (42.0%), added salt (35.5%), and bread wholemeal (6.1%), respectively. The major sources of potassium in these diets were the legumes (41.0%) followed by white breads (11.3%) and fruits (9.1%).

### 3.9 Agreement between urinary excretion versus dietary intake for salt and potassium intakes

**FIGURE 3:**
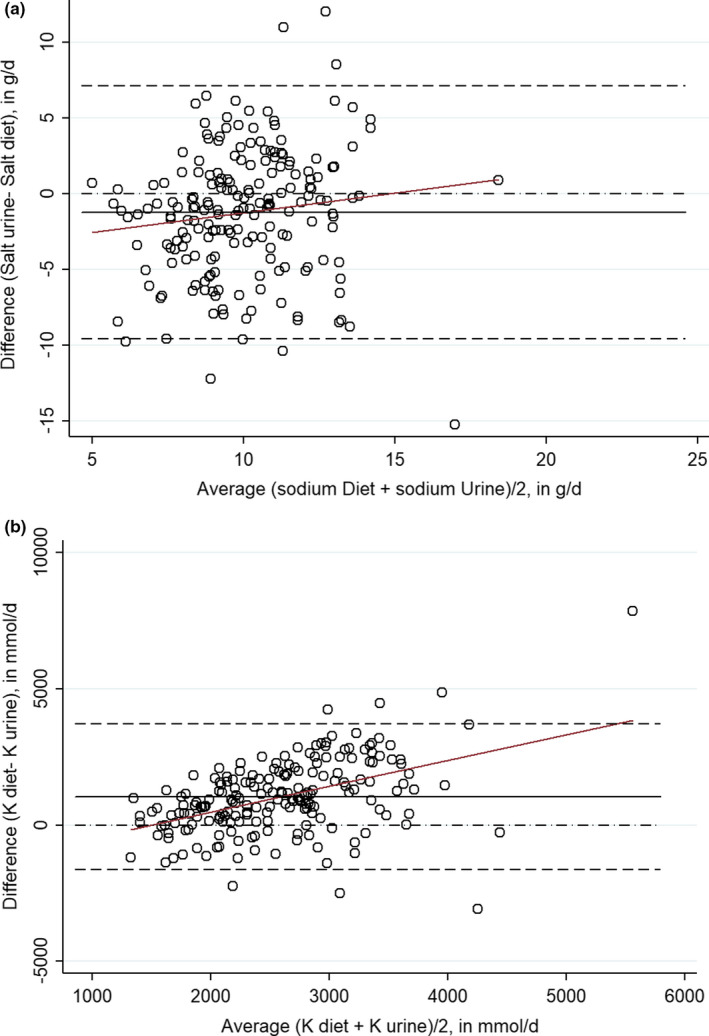
Bland–Altman analysis for agreement of the two methods for assessing sodium (a) and potassium (b) 24‐hr urinary sodium excretion and 24‐hr dietary recall. For women; for Men. Plot against (x‐axis) describes the differences between the two methods are plotted against the averages of the two methods. Plot differences (y‐axis) expressed as crude difference of the values on the axis (i.e., proportionally to the magnitude of measurements). Draw line of equality is used for detecting a systematic difference. Draw lines for 95% confidence interval (95% CI) of mean of differences: The 95% CI of the mean difference illustrates the magnitude of the systematic difference. Draw regression line (red color) of differences is used to detect a proportional difference

The comparison between mean salt intake (Figure 3) measured by 24‐hr dietary recall and urinary excretion showed a significant difference of −1.2 g/d (95% CI: −1.8 – −0.6) with higher values delivered by the 24‐hr dietary recall method (*p* =.0001). The Passing Bablok regression showed a slope of 1.5 (95% CI: 1.1 – 2.0) and an intercept of −5.9 (95% CI: −11.8 – −1.8).

The median difference of 1,030.2 mmol/d (95% CI: 837.3 – 1,223) corresponds to a significant difference between both methods (*p* <.0001). The Passing Bablok regression showed a slope of 3.0 (95% CI: 2.2 – 4.4) and an intercept of −2938.7 (95% CI: −5664.5 – −1309.8).

## 4 DISCUSSION

### 4.1 High salt intake among Tunisian adults

To our knowledge, there are few studies conducted for assessing salt intake among Tunisian population but none of the studies tried to use the sodium excretion among randomly selected participants at subnational or national level. According to our study, Tunisian adults are exposed to elevated doses of salt intake (8.1 ± 2.7 g/d or 138.3 ± 46.5 mmol/d) based on 24‐hr urinary sodium excretion. Furthermore, dietary survey method has revealed a higher value (10.6 ± 2.9 g/d). Almost, 87.1% of subjects had > 5 g/d of salt intake, while 26.3% consume > 10 g/d which is below the prediction made by Mason et al. (2014) of 14 g/d (Mason et al., 2014). However, the intakes may fluctuate significantly across the living area (urban vs. rural) and regions. A study conducted among Tunisian obese (*n* = 56) showed a daily salt excretion of 11.0 ± 4.6 g/d which is higher than that reported for the same BMI category in our study (8.4 ± 2.7 g/d) (Amrouche et al., 2017). Tunisian values are similar to those reported from Spain (11.5 vs. 8.4 g/d) (Ortega et al., 2011), lower to those reported among Lebanese men (12.5 g/d), and lower than what Lebanese women showed 7.5 g/d (WHO, 2015). Moroccan adults’ population (7.0 g/d) seem to have less salt intakes than what we found in our study (Derouiche et al., 2017). In general, salt intake in most of the European countries is higher than the assessed Tunisian adults in the following study. At the time of the study, the comparison of salt intake among Tunisian adults with their homologous in MENA (Middle East and North Africa) region countries, based on urinary sodium excretion, would be biased as available data are derived from dietary assessments.

### 4.2 Reduction of salt intake policy in Tunisia: where do we stand?

In the present study, we have investigated the main contributor food groups to Tunisian adults’ sodium intake using the 24‐hr dietary recall. We found that bread had a decisive influence over sodium intake, contribution of 32.7%, irrespective of gender or socioeconomic status. During the last national nutrition survey conducted in 2005, bread was the main contributor to sodium intake (TAHINA). This fact consolidates the used approach of the bread reformulation (lowering salt content) for a sustainable reduction of salt intake. The bread reformulation was a common approach over the major countries of the MENA region to reduce salt intake (Alhamad et al., 2015). Bread contributes to non‐neglected amount of daily intake of salt ranged from 14.6% in Egypt to 53.9% in Morocco (Alhamad et al., 2015). The lowest salt content in bread is reported in Jordan (average of 0.42 g/100 g of bread), while Morocco bread still contains higher amount of salt (1.47 g/100 g of bread) (Alhamad et al., 2015).

The experiment of salt reduction in bread has already begun in the city of Bizerte (pilot region) since 14 February 2015. At first, an investigation within 22 voluntary bakeries (in total 45 bakeries) was conducted in order to determine the average amount of added salt in bread in the different bakeries (WHO, 2015). Results showed that the percentage of added salt was about 1.6 g/100 g of flour. Bakers were instructed to decrease the quantity of added salt by 20% every six months during a period of 18 months. After subscription of the baker to the “program of reduction of salt, sugar, and fat”, weekly sampling was made during the first 3 months and then once per month to follow the evolution of salt concentration in bread over 3 years. The salt content was reduced successfully by of about one third (*detailed data not shown*).

Salt contributes significantly to the bread taste, so it was supposed that its reduction may alter the flavor perception. Thereby, we suggested that the reduction should be operated over a long period, so that the consumers do not detect this salt reduction (Liem et al., 2011). If necessary, saltiness enhancer (e.g., glycine monoethyl ester and L‐arginine) and salt replacers (e.g., potassium chloride) were proposed to increase saltiness’ perception (Kilcast & Ridder, 2007).

### 4.3 Inadequate potassium intake among Tunisian adults

In our study, daily potassium excretion is under the recommended lower tolerable limit by the WHO and only 10.3% has achieved an adequate intake. Potassium excretion was not associated with overweight or obesity status denoting that this biomarker might not reflect a healthy lifestyle. Major dietary sources were legumes (41.0%) and dairy products (13.4%), while low contribution was found for fruits (9.1%) and vegetables (0.6%). The best approach to improve potassium intake is by increasing the fruits and vegetables consumption (He & MacGregor, 2008). The mean sodium‐to‐potassium ratio was 2.4, more than twofold greater than the desirable level of 1.0. It is essential to recall that an adequate intake of potassium is believed to have a beneficial effect on human health (e.g., reduce blood pressure, antioxidant effect, and glucose tolerance) (He & MacGregor, 2008).

### 4.4 Methodological and biological aspects of electrolytes monitoring: strengths and limitation

#### 4.4.1 Use of 24‐hr urinary sodium and potassium parameters

In order to limit selection bias, random sampling was used to select the participant. The one‐day 24‐hr urinary collection method was used to accurately evaluate sodium and potassium intake of individuals (WHO, 2012, 2013, 2016). This collection method is shown to be the most reliable indicator for the monitoring vs. other methods (i.e., causal urine sample or dietary assessment).

The assessment of the completeness of 24‐hr urine collection is still challenging, and several approaches have been developed by researchers (Elliott & Brown, 2007). Unfortunately, the para‐aminobenzoic acid was not used for the validation of 24‐hr urine completeness; however, errors were minimized by using several survey methods of control (e.g., time, urine volume, creatinine excretion).

Due to within‐ and between‐subject biological variations (28.7% and 16.7%, respectively (Ricos et al., 1999)), one‐day 24‐hr urinary sodium determination may not reflect the average consumption. In addition, antidiuretic hormone and aldosterone are subject to seasonal variation with a markedly elevated concentration during the summer. It is important to highlight that any constant change in sodium intake can take at least 3 days to be reflected in urine and to rich “steady state” (Lucko et al., 2018).

The indirect potentiometric method was used to measure urinary sodium levels. A negative bias might be reported in case of higher amounts of glucose excreted in urine (Goyal et al., 2016).

#### 4.4.2 Dietary assessment of sodium and potassium intake

The dietary survey (3‐day 24‐hr dietary recall) was the second approach used to assess sodium and potassium intake from diet. The main limitations of this method were as follows: inaccurate or incomplete food composition tables and missing data (Rhee, 2015), variability in added salt during cooking (in house or in fast‐food) (Elliott & Brown, 2007), variability sodium content in foods depending on the season (Moreno‐Rojas et al., 1994), and possible losses of minerals during cooking processes (Kimura & Itokawa, 1990).

We have found that the dietary survey method tends to overestimate the sodium intake in comparison with the sodium intake estimated by 24‐hr urine collections. This observation is in line with those reported by Zhang et al. (2013) and Peniamina et al. (2019). Contrastingly, several other reports (Espeland et al., 2001; Leiba et al., 2005; Schachter et al., 1980) showed that the dietary assessment methods underestimated the intakes of sodium. Even if this approach demonstrates several limitations, it is considered as an unavoidable tool to address public health concern and to assess the main food contributors of the daily sodium and potassium intakes.

## 5 CONCLUSION

Our study demonstrates that a high sodium intake and inadequate potassium intake were found among adult population living in urban city. According to the World Health Organization, a Tunisian strategy was launched aiming to the reduction of salt intake. Based on the following data, the reduction of sodium content in bread was adopted as the main procedure. To overcome iodine deficiency, Tunisia authorities have adopted the universal salt iodization and salt is the main iodine intake contributor (Doggui et al., 2017). Reducing salt intake below 5 g per day may imply a revision of salt iodization threshold (Zimmermann, 2014). Authorities should be aware about that fact, and a closely monitoring of both iodine and sodium intake consumption should be conducted during the following years.

## 6 ETHICAL REVIEW

The protocol of the survey was reviewed and approved by the Ethics Committee according to the guidelines laid down in the Declaration of Helsinki on Human Research of the National Institute of Nutrition and the Tunisian National Council of Statistics (visa n°8/2014). After being thoroughly informed on purpose, requirement, and procedures, all participants gave their free informed consent.

## CONFLICT OF INTEREST

The authors declare that they have no conflict of interest.
